# Work-Life Balance in Great Companies and Pending Issues for Engaging New Generations at Work

**DOI:** 10.3390/ijerph16245122

**Published:** 2019-12-15

**Authors:** M. Isabel Sánchez-Hernández, Óscar Rodrigo González-López, María Buenadicha-Mateos, Juan Luis Tato-Jiménez

**Affiliations:** Business Organization and Sociology Department, School of Economics and Business Administration, University of Extremadura, 06006 Badajoz, Spain; orodrigo@unex.es (Ó.R.G.-L.); buenadic@unex.es (M.B.-M.); jltato@unex.es (J.L.T.-J.)

**Keywords:** work-life balance, best companies to work, web recruitment contents, new generation at work, millennials, generation Z, generational diversity

## Abstract

The changing nature of employment and work causes new demands in society, such as work-life balance, that has emerged in labor relations as an important aspect of a healthy work environment. In this context, Best Companies to Work for are a reference in caring for their staff, and it is well known that new generations—that frequently use the Internet to be informed—are making their decisions as job seekers by checking and comparing corporate websites. In order to learn from the best companies, but also to discover what could be improved by identifying the gaps, this study observes the current work-life balance practices in the last Best Companies to Work for awarded by Fortune. The main contribution of this work is the development of a weighted index for benchmarking purposes considering the preferences of new generations at work. The study demonstrates that the best companies still report low levels of work-life balance information. The main implication drawn from the study, due the requirements of new generations at work and the rapidly emerging field of e-recruiting, is the need for human resource departments to fit work and personal life in a fluid way, while maintaining a healthy balance. It is also recommended for companies to improve their disclosure of work-life practices on line for attracting talent from Millennials and Generation Z.

## 1. Introduction

The changing nature of employment and work is having a profound effect on how talent is managed [1]. Increasingly employees are interested in flexibility and freedom in their workplaces [2]. In this context, work-life balance (WLB) has emerged as an important aspect of a healthy work environment [3], and as a new strategic human resource policy [4], with great repercussions for workers and their families, organizations, and society as a whole. For instance, employees who are distracted by WLB pressures end up costing companies in several ways, from lost productivity, absenteeism and disengagement, to medical expenses, among others, and the impact on business revenue [5].

WLB is a current concept that may be understood as the existing relationship between work and personal life, but with a great impact on factors such as health, absence of stress, well-being, quality of life, organizational performance, and sustainable human and social development, among others. For instance, the European Foundation for the Improvement of Living and Working Conditions asserted that a deficient WLB lowers employees’ quality of life [6]. In the same line, the Commission on Social Determinants of Health and The World Health Organization has highlighted that health equity requires a healthy WLB for all [7].

Nowadays the proactive promotion of the WLB by organizations has become more important than ever because new generations arriving at work claim that work environment should be fluid and flexible. In addition, meeting the needs of the new generations of employees undoubtedly leads to a happier workforce who will then share their positive work experience with others [8], having positive consequences for consumers or clients, and for the organization as a whole [9]. In this respect, organizational tools such as child care services, extended and flexible parental leave schemes, or support to single parents, among others, may increase reconciliation of work and domestic life [10].

The concern for the integration of different parts of people’s lives, such as work and family welfare is not new. As Lewis et al. argued [11], in the 60s the focus mainly was on working-mothers [1], but from the 80s the work-life conflict and its relationship with stress and burnout already generated concern and research.

In the 90s appeared the most current WLB family friendly policies that have been growing since then, pointing out that the management of WLB is an issue increasingly recognized as strategic for organizations [12,13] because WLB practices reduce costs in human resource management by recruiting high-quality professionals and retaining the best employees [14,15]. WLB is also highly significant for employees [16], with impacts on the public health [10], and is clearly necessary for a sustainable human development [17].

The concept of WLB, despite its simplicity of understanding, is a term that is still difficult to conceptualize and to measure [18]. However, and fortunately for workers, WLB is becoming a trendy topic in recent years [19] in the personal sphere and also in the organizational context [20]. Although the sectors studied are diverse, recent studies on the topic focus on health [21,22,23,24], transport [25], or education [26,27]. The study of WLB is specifically prominent in the field of public health [3]. There is evidence of employees reporting a poor WLB reporting more health problems [10]. Further, a poor WLB can be seen as a work stressor, and academic literature also shows a relation between work–life imbalance and stress responses, such as high blood pressure, heart rate, or cortisol levels [28]. A recent study, more focused on the impact on work, indicates that WLB is an organizational lever reducing stress and willingness to exit for employees of the organization [29]. Moving to a rich WLB, literature indicates that WLB helps in the development of psychological availability and augmenting employee positive energy [30], and highlights that the satisfaction level with the WLB plays a role in supporting mental health [22]. Others works have indicated that WLB has a positive effect on job and family satisfaction [31], and recently it has been proved that a positive correlation exists between job satisfaction and WLB [24].

The new generations really value WLB for the importance attributed to fitting work and personal life in a fluid way, while maintaining a healthy balance. Different studies indicate that Millennials and Generation Z understand the need of the WLB and give it greater importance than previous generations at work. ManpowerGroup [32] shows that among the main priorities for jobs of the Millennial generation, along with salary, job security, and the work environment, are factors related to the promotion of a suitable WLB, specifically vacations and free time, and work flexibility. Following Twenge [33], it is possible to affirm that new generations at work clearly differ from other previous generations. For example, Baby Boomers mainly sought stability and security in their work, and Generation X gave more importance to work relations over other variables.

In this new scenario, the purpose of this paper is to study the WLB programs that the best companies are offering at the moment. With this aim, we have chosen distinguished companies with the certification Best Place to Work for (BPW), that annually and publicly recognizes outstanding work cultures. The list of the 100 BPW is elaborated by Fortune Magazine and is internationally recognized. Based on this ranking, the research question was what should be learned from the WLB programs of the BPW to attract and retain talent, linked to the fact that new generations of employees are gaining presence in the company staff. To answer this question, the study focuses the attention on corporate webs, as crucial contemporary means for recruitment and as a relevant showcase where organizations can show how to interact with job seekers and employees [34]. We put the attention on information related to the workers WLB tools. The purpose was to examine what practices are being mostly applied in distinguished companies and their importance for new applicants.

## 2. Theoretical Background

### 2.1. The Irruption of New Generations at Work: Millennials and Generation Z

In recent years, some authors dare to state we are changing to a new era. In any case, the world is changing dramatically [35,36,37]. Boundaries are disappearing and aspirations are changing. People do not simply expect a job with a balance between work and private life, but even a harmonious integration between life and work [38]. In fact, current human resource managers have the view that important inter-generational differences exist among workers and that these differences provide challenges in effectively managing workforce [39].

Different sources show slight changes in the timing of the generations but broadly generational limits are the following. Gen Silent are those born before 1944. Baby Boomers are people born from 1945 to 1964. Generation X are considered those born from 1965 to 1980, but Millennials are those people born between 1977 and 2000. There is no consensus regarding the date but all the authors stress the fact that the technological coordinate stands for a distinctive feature. Generation Z, or Gen Z, is defined as the generation born between 1995 and 2004, which currently puts them between 15 and 24 years old. This is a generation of true digital natives. Growing up constantly connected to their smartphones, 42% of Gen Zers say they interact more with their phones than they do with people [40]. They also have less guilt about using technology than their older counterparts. Most studies state Gen Z is ready to work hard and optimistic about the future [40].

### 2.2. Work-Life Balance for Fulfilling the Needs of New Generations

Basically WLB is a concept that reflect the balance between work, family commitments, and personal life [41]. Kelliher et al. [42] point out that where achieving a satisfactory WLB is normally understood as restricting one part (usually work), to have more time for the other. The growth in attention to WLB evidences that it is an issue that worries many, from the spheres of the individual and of organizations, and society in general. Millennials and Gen Zers really appreciate relationships and personal time, so WLB is highly valued too.

In the WLB literature three basic dimensions are generally studied: social, organizational and personal. In this regard it should be noted that there is a wide range of conditions with a relation on the WLB of employees. Further individual and organizational characteristics, and cultural and political contexts can also have influence on the WLB [10]. This perception-centered concept is very relevant, that points to the WLB as a holistic conception, which is the subjective value of balance between work and life [43]. This subjective value, associated with the individuals’ perceptions of their own situation in every moment, makes it complicated to establish objective indicators for WLB and its consequences.

In this context, and even acknowledging other alternative approaches, in this work we assume that the current approach for conceptualizing WLB falls within the situationist perspective, in line with Reiter [44] and Kalliath and Brough [45], and according to the taxonomy of ethical ideologies and positions of individuals by Forsyth [46]. The situationist framework for understanding WLB has being considered appropriate because it evaluates the balance within the context for each employee at work. This framework recognizes the contribution of both the environment and the individual, and the complex interplay between them and consequently it involves making optimum choices for individuals. WLB approaches framed from this position have focused on a fitting definition of balance for any employee depending on their specific personal contexts [47,48,49].

Under this approach, the WLB concept depends on the people’s characteristic and their perceptions. In fact, there are studies that indicate that WLB programs show indirect effects to help employees improve their well-being conditioned by individuals’ positive attitudes and their life coping strategies [50]. For instance, the WLB has been demonstrated to have positive relation to the employee self-efficacy [31]. Other study shows that the emotionally intelligent employees are better in managing their WLB and are more satisfied with their job [24].

To sum up, part of the research on WLB tries to check employee relationships with different variables. Among others, WLB is related to health, development of psychological availability, and augmenting employee positive energy, stress, and rotation, employee well-being and satisfaction, and sustainable human development. In the actual context it is essential to research and know in practice the current role of organizations offering new generations at work the expected human resources management measures that promote adequate WLB.

### 2.3. Common WLB Practices

Organizations offer a pool of practices to implement WLB. Generally, there is no single accepted definition of what constitutes WLB practice. They are defined as institutionalized structural and procedural arrangements, formal and informal, that enable individuals to easily manage the conflicting worlds of work and family leaves [51]. Another practical definition of WLB practices refers to work options that provide flexibility in terms of where and when work is conducted [5,52].

The term WLB practices usually refers to support for flexible work options, dependent care (child and elder), and personal, parental, and family leaves [53]. In this sense, WLB support provided by employers includes, for instance, flextime, job sharing, moving from full-time to part-time working, compressing working hours, home working, or only working in the school term, among others.

We can group in flexible work arrangements practices associated with the flexibility of the working schedule, the number of working hours and remote working. The most common WLB practice identified in the academic literature is flexible scheduling [4] such as flextime and flexplace, which permits workers to vary their start and finish times of work, assuring that they complete the number of allocated acceptable working hours [54]. Flexible hours can bring employees the opportunity to control the work schedules adequately according to their needs reducing work-life conflict [55].

In the same line, other practices are starting to be relevant for WLB. For instance, the use of a compressed workweek, an arrangement that allows employees to work longer shifts in exchange for a reduction in the number of days in their workweek cycle [56]. Remote working is also associated with the flexibility of place to work. Specifically, tele-working is a controversial practice [57] of working at home or travelling while interacting with the office [56]. It mainly refers to the use of laptops, videoconferences, and the possibility of having the Internet at home to develop the labor tasks and duties from home. Companies might give the chance to some employees to do it regularly. With this practice, the employee is potentially in a better condition to manage his/her time to balance personal and work life. For example, tele-working eventually permits taking care of relatives at home while someone is working, and it might reduce the work-life conflict [58,59]. The access to resources for part-time arrangements as one way of achieving a WLB that can also allow those with health problems or limited time to participate in the work process, develop their skills and obtain working experience by even being elsewhere apart from the work station [60].

In general terms, flexible work is associated with more positive work attitudes [61] and is believed to influence positive attitudes because it symbolizes the organization’s concern for employees [62]. The availability of flexible work arrangements can also increase the perception of psychological control, which can help to alleviate the conflict between work and personal life [63]. Conciliation practices provide workers autonomy to decide how they wish to balance the work and life spheres [64]. In general, flexible work arrangements are highly valued for employees in order to experience a richer WLB.

In line with the aforementioned relatives’ care, Lourel et al. [65] also pointed out that any organizational support aimed at facilitating care services such as childcare and elderly care is another WLB practice. In addition, WLB practices related to family and parental leave are sabbaticals for employees. A sabbatical means the employee is taking unpaid leave for a determined period of time such as weeks or even months for personal issues including projects for change of careers or personal development, and also for care family [66]. However, most of the times the legislation adopted in different countries grants the individual employee the right to request leaves, especially in the cases of the provision of child and elder care services (for example family leave measures such as maternity, and also paternity, are common in most developed countries). It is a paid leave to care for dependents in an emergency. In the same line, child and dependent care refer to the help to comply with the demands of home and the commitments and obligations outside work [67].

**Figure 1:**
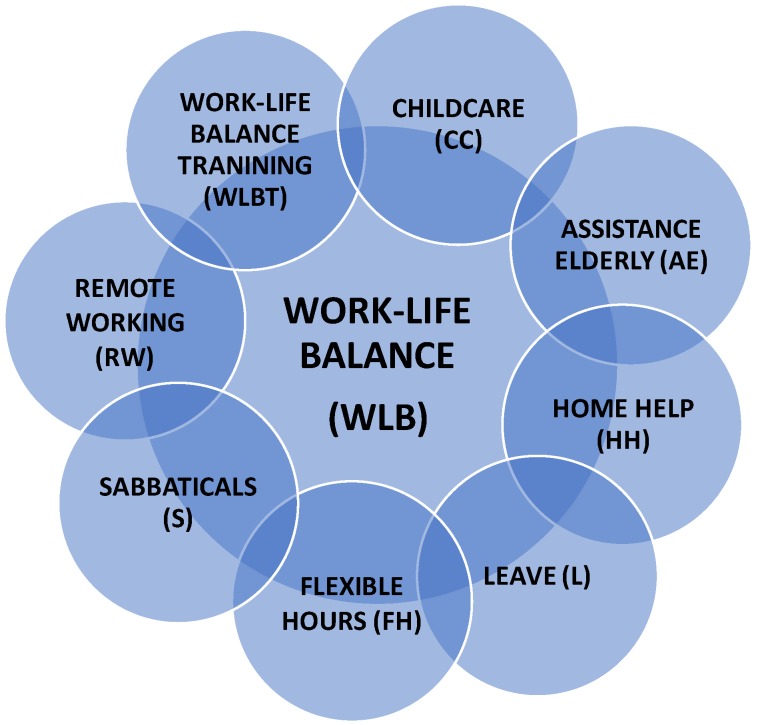
Common work-life balance practices.

In addition, contributing to WLB we highlight any kind of home help such as restaurants at work, assistance for shopping or dry cleaning, assistance for the elderly (i.e., medical services or money) and child care (i.e., kinder garden at work) [68]. Finally, and gaining importance in big companies, we highlight training and education for workers and their families about WLB. This practice is desirable because it could be highly beneficial for obtaining better results from conciliation policies. Providing workers who can control their working hours with information on healthy and social work scheduling would help them to minimize unintentional social impairments due to their work hour choices [55]. WLB training must be technical and it must also include conflict management and stress management. To resume the organizational support for WLB, Figure 1 as follows represents the most common WLB practices.

### 2.4. Work-Life Balance: A Challenge for Companies in Present-Day Society

Following Lyons et al. [69], we agree that generational effects are inherently confounded with age and historical period effects. We share the view of the generational theory [70,71,72], which posits that society alternates between a cycle of growth, conformity, decay, and divisiveness, and that each cycle is driven by the changes in the values and attitudes of each new generation. However, we also share that it is not necessary to disentangle the generational effects of each period for understanding the phenomenon of generational differences [69].

This view fits very well the aforementioned situationist framework for understanding that the WLB concept depends on the generation’s characteristic and their perceptions. Consequently, those companies that today try to understand the huge upcoming employee segment entering in the labor market will gain a large competitive advantage if they show a good image and get a great employer brand, through fitting their WLB practices to the new talent.

Thus, every generation is shaped by its circumstances, and the new generations entering the labor market—Millennials and Gen Z—are not an exception. They are no less ambitious than previous generations, but their priorities have changed and evolved. Having children, buying homes, and other traditional signals of adulthood considered success markers do not top their list of ambitions. Related to work expectations, on the one hand, as stated by the literature concerning this topic [73,74], Millennials desire a high level of autonomy, and do not just work for a paycheck because they want a purpose. Millennials also expect a high level of coaching and feedback and desire a high level of customization, because it is not just their job, it is their life. In addition, Millennials prioritize three things when choosing where and how to work: money, job stability, and free time. They also value positively and enjoy a flexible work environment, learning new skills, and having the opportunity to grow in the company [75].

It is relevant to remark that Millennials are characterized as having high levels of self-confidence and self-reliance. They are independent, individualistic, and socially active, and like to work in teams [76]. For all these reasons, the most important information for Millennials jobseekers and potential candidates for process of recruiting is the importance of time flexibility, together with the benefits of the position. In this sense, all the expectations we cited above determine what they are looking for.

In between many wishes, we want to focus on WLB, and Millennials need to find balance in their personal and working lives [77,78]. Millennials require a flexible work schedule as well as a comfortable environment [79], and then they will flourish. They will challenge the status quo by showing adaptability and openness to change. Some companies already understand these needs and are offering a flexible working schedule, volunteering work opportunities, extra benefits, and by encouraging creativity and innovation [80].

On the other hand, Gen Z talent brings a change in attitude and perspective reflecting a return to more traditional workplace values such as the desire for a clear career path and stability. Nevertheless, these potential employees are not old school, they bring a future-forward outlook, in the form of digital skills and mindset, to any employer [81]. They express preferences for large firms standing in contrast to the recent past (47 percent went to work for small businesses versus 23 percent at companies with more than 1500 employees). They eschewed large employers in favor of digital disruptors with a startup culture, in which young graduates gravitated toward valorizing entrepreneurship [82]. The most significant generational difference stems from the emphasis placed by young people on family and leisure. Gen Z workers expect flexibility on the part of the company they work for to help them maintain WLB, one of their top concerns. They also expect training and mentoring, as well as meaningful, challenging work and a clear skills paths.

A challenge for companies seeking a stable workforce is that 49 percent of Millennials would quit their current jobs, if they had a choice [83]. The main reasons to leave a company are dissatisfaction with pay, and lack of advancement and professional development opportunities. Certain characteristics can appeal to Millennials and make them want to join or stay with employers, but the lack of those characteristics may not necessarily drive someone to leave. Employers must wrap all the benefits looked for by Millennials into an engaging, tailored employee experience, to reap the results [84]. Unlike millennials, early indications reveal Gen Z have a willingness to stay with one company [73]. Arguably, the culprit of high Millennial and Gen Z turnover is low engagement. It has been found that 84 percent of the generation is not emotionally and behaviorally connected to their jobs and companies [73]. With just less than one in three Millennial employees engaged, companies have a lot of work ahead of them in retaining this talent. However, employers have to begin to take leadership styles fitted to the expectations of Millennials and Gen Z and workplace practices will need to adapt and evolve to meet the needs of younger generations. For that purpose, many of the engagement and retention drivers for these new generations have to be centered around financial rewards and motivation, as well as programs coping with stress and finding a good WLB [85]. According to this, having a healthy WLB it is a very important issue for new generations, where 15% of male and 21% of female employees would accept a reduced pay and even slow down or lose promotional opportunities if it meant they could work fewer hours [74]. As specifically related, Gen Z is less worried about WLB (38% say this is the most important matter at the job) than Millennials (47% say this is the most important matter at the job) [86]. Another important issue is that WLB is no longer primarily a women’s issue. It is a generational and a societal concern [87]. For now, however, schedule flexibility remains a slightly higher priority among female candidates.

Considering the above, organizations should emphasize their focus on WLB and what they offer employees from new generations to help them reach their physical, community, and social purposes, and financial goals. Companies should also publicize their flexible work arrangements, highlighting flexible scheduling and work-from-home options. Millennials’ and Gen Zs’ preference of online job searches [88,89,90] underlines an obvious need for companies to make their online job opportunities easy to find, user-friendly, and visually appealing. Their websites should also include compelling content related to what the organization offers employees and what differentiates it from the competition.

## 3. Method and Procedure

### 3.1. Research Environment: Best Places to Work

To approach the WLB practices that the best companies currently offer to capture and retain talent, in this work we have chosen distinguished organizations for having good human resources measures that make them deserve a distinction as good employer, employer of choice, or BPW [91]. In order to attract talent, it is important to properly communicate what companies offer to new jobseekers. Organizations can use several classic approaches and means to spread messages related to the company’s brand or employer branding [92,93]. Following an employer branding strategy allows a differentiation that can be very advantageous for organizations. As noted by Ambler and Barrow [94], employer branding is a strategy in which employers can distinguish themselves from others, offering a package of psychological, economic, and functional benefits to employees as it is clear that the benefits derived from having a best employer workplace brand gives an organization a competitive advantage [95].

When the brand concept is applied to human resources management to attract potential employees, it is called the employer’s brand [96]. The brand as an employer of choice allows the organization to distinguish itself from the competition and develop a recognizable identity, through practices that are perceived as desirable in employees and in the general public [95]. It is important to note that both researchers and professionals point out that the implementation of the best human resources practices that support and reinforce a better employer culture is a good investment [97].

To have an adequate brand of the workplace has been increasing gaining importance in recent years, since among other things it has been associated with positive effects on the organization that uses it properly. In fact, some of these positive effects, according to Dell et al. [98], are to generate a competitive advantage, help employees internalize the organization’s values and help attract and retain employees. The employer’s brand helps communicate the reasons why you might like to work for that particular organization and what the organization represents and is a unique value proposition for potential and existing employees [99].

An increasingly powerful form of brand media is found in the popular annual surveys on best employers [97]. These companies have a recognition that differentiates them and indicates them as companies especially concerned with their staff. The BPW in the world represents the companies that are leading this new era of inclusive cultures, with leaders that motivate and inspire people to grow on their own and directly impact the company results. Nowadays, corporate web pages, and more specifically recruitment web pages, can be used to communicate the most attractive human resources practices that the company uses. On the recruitment websites, those practices can be disseminated and can help to project a differentiated image for employees, potential employees, and the general public.

For a company, to be considered as a BPW is a differentiated distinction about their concern for employees that reinforces their brand as an employer. As a result, considering that the new generations of employees use the Internet to get to know job offers, it is very important from the point of view of organizations to adapt the ideas of branding from marketing and communications, and applying them to the recruitment and selection process, and to develop and communicate powerful value propositions about benefits for employees [100]. In the previous Section we showed the practices related to WLB that contribute to create and develop the employer’s brand in leading companies as employers of choice. In the following Sections we will perform our analysis by incorporating the preferences of the new generations, to have a realistic picture of what is happening.

### 3.2. Research Design: Methods, Data Collection, and Samples

A study was performed of the current BPW, focusing the attention and quantifying the web section devoted to careers, with concrete information related to good practices affecting WLB. The list of organizations was gleaned from “The Fortune 100”, showing the 100 best companies to work for at the moment, a list of companies ranked by Fortune magazine. We used the qualitative method and the content analysis for collecting data from the corporate web sites in a deductive approach [101]. Initially the URL for the 100 BPW by Fortune were identified. Researchers checked if websites had a recruiting web and explored whether they published the benefits to employees or not. Given that 31 organizations were not providing information related to our research purpose they were removed from the study. Once identified, the 59 websites were to be deeply analyzed, then, a three-step coding process was followed.

Firstly, and according to the previous literature review, the measures that favor WLB were Remote working (RW), Sabbaticals (S), Flexible hours (FH), Leave (L), Home help (HH), Assistance for the elderly (AE), Child care (CC) and WLB training (WLBT). Later, the initial six sites from the list were coded by using our ad hoc coding approach. The aim was exploring practices or programs directly related to WLB. Given the number of websites, the variety of types of organization as goals and sectors, and the need to ensure reliability between researchers (as coders), this stage was done as a training that would ensure that all the authors followed a similar procedure and would filter possible misunderstandings. In this moment, coders met to compare their coding and discuss when there were coding differences. Once total agreement was achieved between coders, the same protocol was used to collect, systematize, and analyze the data of the rest of the websites.

At the last stage for coding, the BPW websites were randomly divided between the coders. Data was collected in the form of specific practices or tools related to WLB, in which the emphasis was placed on an in-depth understanding and description of the actions found, and not only on determining frequencies. Because of that, and also considering the reduced number of websites and primary codes, the coding process was performed on a traditional way, without the support of any computer-assisted qualitative data analysis.

Additionally, we used the BPW Fortune website to study the generational diversity of employees, recording the proportion of employees belonging to the Z, M, X, B, and S generations. From the total, nine companies were excluded as they do not show this information. Considering that different studies show slight changes in the timing of the generations, in this work we have chosen the temporary location followed by Fortune to offer the data of the templates of each of the BPW certified companies. The proportion of employees belonging to the M and Z generations in the 100 BPW list is 42.9% (39.3% M and 3.6% Z).

The study applied two complementary analyses: content analysis of websites belonging to BPW companies and a survey about preferences at work for Millennials and Gen Z. The conclusions were drawn based on the results of these analyses. Thus, data gathered served for a twofold purpose. Firstly, secondary data gathered from companies served for discovering whether the current BPW are concern about WLB. Secondly, primary data gathered from Millennials and Gen Z in our study served for understanding how BPW have to modulate their WLB programs to fit the new generations at work according to the theoretical situationist framework.

As the first step, we performed a descriptive analysis for characterizing WLB practices at BPW for attracting talent in their websites. The final sample had 59 companies of the initial sample of 100 BPW that are those that had information on their web pages related to WLB as a tool for attracting and retaining talent. The analyzed companies belong to 12 activity sectors with different representation in the BWP ranking as follows: Financial Services and Insurance (N =12); Information Technology (N = 11); Professional Services (N = 10); Health Care (N = 5); Retail (N = 5); Manufacturing and Production (N = 4); Construction and Real Estate (N = 3); Biotechnology and Pharmaceuticals (N = 2); Hospitality (N = 2); Telecommunications (N = 2); Transportation (N = 2); Advertising and Marketing (N = 1).

The second step was to develop and calculate and ad hoc self-administered questionnaire in Google Forms for a convenience sample of Millennials and Generation Z, conformed by finalist students at the university to which the authors of this study belong. This course of action was selected considering the sample was formed by individuals close to their first job. To ensure the non-existence of biases in the sample, individuals were previously instructed about the meaning of WLB, the common practices, and their potential benefits. A total of 131 finalist completed the survey. The average age of the participants was 20 years old, 42% were men and 58% women.

## 4. Results

The description of the areas that were coded and examples of what counted in each category are shown in Table 1.

**Figure 2:**
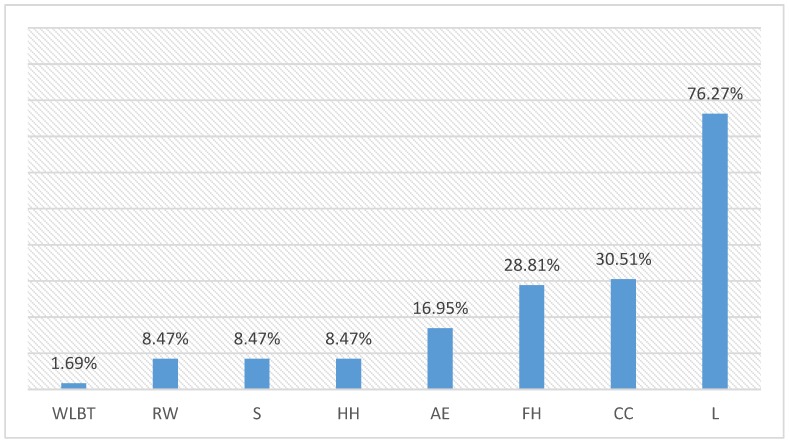
Work life balance (WLB) practices in Best Places to Work for (BPW) organizations.

In addition, Figure 2 shows the relative presence of each WLB practice in the sample of BPW organizations considered. The results show that there is a relatively low presence of WLB practices when considering that these organizations are the current BPW. Except in the case of leave opportunities, offered by 76% of the companies, the rest of the practices evaluated were not offered by half of the companies. The following practices in importance were child care, offered only by 30% of the companies, followed by flexible hours, and elderly assistance.

To complement the descriptive analysis of secondary data from companies, we performed a quantitative analysis of the generational diversity presented in the current BPW staff, with special interest in the proportion of the youngest employees. As it is shown in Table 2, new generations M and Z have a considerable presence in BPW in average, although there is high dispersion on data.

Our data from companies also served for creating a weighted index for benchmarking purposes, using as weights primary data coming from our survey to Millennials’ and Gen Zers’ preferences at work related to WLB alternatives. Given the different aspects included in WLB programs, and the fact that not all of them will be equally appreciated for M and Z job seekers, a weighted index was created in order to compare the organizations:

(1) $$Index=\overset{n}{\underset{i=1}{\sum }}B_{i}\times V_{i}$$

where *B_i_* is the benefit offered, and *V_i_* is the weight provided by M and Z job seekers through the ad hoc survey. The most preferred was FH (4.21) followed by CC (4.05), L (4.04), RW (3.96), WLBT (3.89), AE (3.5), S (3.18), and finally, HH (3.04). Once data were collected and analyzed, for an easier interpretation, the index was standardized (Zindex). Thus, a score of 0 would indicate that the organization is in the average; the positive values would indicate a level of WLB better than the average, and negative values would indicate that these companies are below the standard. Using the WLB Zindex measure, Table 3 shows the list of the Top Index WLB Ranking.

## 5. Discussion

Our results have several implications for replication studies about WLB using qualitative research, specifically regarding managerial practices through the utilization, or own construction, of the WLB index. In our study, all sectors are represented in the list of the Top Index WLB Ranking, except Telecommunications, Hospitality, Real Estate, and Transportation. It is important to note that these last three sectors have all the companies on the list with WLB Zindex below the average. On the opposite extreme, we want to point out the sectors where more than half of the companies have higher value than the Zindex average. In this sense, the only company in the Advertising and Marketing sector (Alliance Data) is above the Zindex average, reaching the third highest value in the total in the list of the Top Index WLB Ranking. The following sectors are Health Care, Professional Services, and Retail (all three have 60% of companies above the Zindex). We want also to remark that Hospitality and Real Estate and Transportation have a low presence in BPW rank and the existing companies are below the Zindex average. Additionally, the case of Information Technology is noteworthy, with 73% of companies with Zindex of WLB below the average.

In the companies analyzed the highest percentage of employees of the Z generation was Burns and McDonnell, belonging to the Professional Services sector, with more than half of the total staff (54% of the total) and the company with the highest proportion of employees belonging to the M generation was Build-A-Bear, from the Retail sector with 81% of M employees. Both companies have a WLB Zindex above average.

The results show that the position of each one in the BPW ranking is not necessary related to being a leader promoting WLB in their web. In addition, job seekers preferences for different WLB programs are conditioning the final position of each company in the Zindex final ranking. For example, the winner of the Zindex ranking was JM Family Enterprises, that was placed 51st in the original BPW list.

We want to remark that interesting WLB examples, to the best in the Zindex, could be used by other companies to improve their own programs. For instance, the leader of the Top Index WLB ranking JM Family Enterprises includes the following: “Salon services: JM Family’s Founder Jim Moran began the tradition of free haircuts for associates during his early days as a car dealer in Chicago. He would bring in barbers for his sales staff to help them look and feel their best. The tradition continues today at Salon 185! The salon offers free haircuts and manicures to associates. (…) Eco-friendly dry cleaning and shoe repair drop off and delivery service”. Additionally, a BPW that is in the Top WLB index is Hyland that spreads the message: “Here, work-life balance isn’t just a buzzword. Feel the energy and support of people driven to make each day count”. Another example belongs to another organization at the top, and moreover the company that has the highest proportion of employees of M generation (81%), that is Build-A-Bear. In its website it appears the following customizing WLB messages with the company: “Honey Days allow you to use your best judgment in the use of available time off, whether it is for vacation, sick days, religious holidays or to attend to personal business (…) Birthdays are a reason for celeBEARation at Build-A-Bear Workshop. All associates are given a day off with pay in the month they celebrate their birthday!” With these good practices we finish the presentation and discussing of results to provide the summary of the study and conclusions, as follows in the last Section.

In general terms, the results show that there is still a low presence of WLB actions and programs, or at least, we have found less actions than expected. It seems that although at the theoretical level the promotion of these measures is justified, and although the new generations value them as important at work, in reality they are not being offered at the necessary level. This is even more serious when, as in this work, companies that stand out for their concern for staff and have a distinction such as BPW are analyzed.

Except in the case of the Leaves that allow employees to have more free time than legally established (Leaves reached a high level of presence in our study of 76%), the rest of the tools analyzed do not reach 50% of presence. Consequently, we can affirm that there are pending issues to be considered in the future to improve WLB programs. The current weight of these WLB measures have to be clearly improved. For instance, CC is only offered by 30.5% of the companies considered as excellent in managing people, followed by FH (28.81%) and AE (16.95%). Sharing the same importance value (8.47%) are the measures of HH, S, and RW. Finally, the BPW commitment to training actions that develops skills for employees to manage their WLB are practically non-existent (1.69%). So much effort has to be done from now in giving relevance to these tools for really attracting the best employees from M and Z generations.

Although the literature reviewed supports the importance of adequate WLB at different levels—at the individual level, with repercussions in the family, and in the organizational and social sphere—there is evidence that a deficient WLB lowers employees’ quality of life (Wallace, Pichler, and Hayes, 2007). However, as we have seen, the new generations of employees are demanding WLB measures that allow them to successfully perform their jobs. The results of this study reinforce the importance of WLB for the youngest, showing that the WLB programs are an important motivator for the new generations (M and Z) at work. This is important for all organizations but also for BPW as this work has quantified the strength of new generations in these companies showing that the presence of the M and Z generations is now near half of the staff (42.9%).

## 6. Conclusions

This study has focused the attention on the study of WLB practices in BPW, organizations that are a benchmark in human resources management. Although there are many researches that support the individual and public benefits of promoting measures that support the WLB, this study has shown aspects that could be improved in managing talent, specifically from new generations at work. Among several benefits derived from the implementation of WLB practices cited in the literature, we highlight they help organizations to attract and retain talent [5]. However, according to the results obtained, we can say that the BPW do not offer, at the moment, the expected and adequate level in its WLB programs, considering the number of organizations showing WLB programs as part of their web recruitment communication.

The research findings contribute to the extant scientific knowledge on the topic with theoretical and practical implications. The study contributes to the academic literature on WLB and generational diversity in three ways. Firstly, by identifying the elements that organizations could offer to design complete and attractive WLB programs. That could have a significant influence on employees’ health, organizational performance and social welfare. In addition, the study also identifies the pending issues in WLB in accredited companies as the best places to work for. Secondly, the study provides new insights into knowledge of the strength of the presence of new generations at work and their preferences and demands. Thirdly, this study is useful for practitioners because some implications emerge from the study.

The practical implications of the study determine its originality and its value-added. It has been shown that Millennial and Generation Z are demanding a good work environment with factors related to the promotion of a suitable WLB, specifically work flexibility. Thus, for benchmarking purposes, the study offers a WLB ranking by considering the studied tools available and the preferences of M and Z generations. In this ranking one organization of the Manufacturing and Production sector has been revealed as leader, JM Family Enterprises. Consequently, this company can be considered a good example in managing WLB for other companies around the world. Thus, other companies have now a reference for checking their WLB practices, trying to improve their programs in order to attract and retain new generations. Thus, our findings can be used by researchers and human resource managers or external professional services providing support to them, to attract and retain the best employees, and to adapt human resources policies to the expectations of the new workers, who already have quantitative relevance in organizations. Benchmarking efforts can be useful for improving personal well-being, health, and organizational development by beginning examining successful experiences, and designing later improved WLB programs.

Although the study has a contribution to the existing knowledge on the topic, it has also some limitations. First, reliability and validity of any content analytic research must be in mind because the study is cross-sectional, partially based on secondary information self-reported by the organizations of the sample in their recruitment webpages. Secondly, limitation is in relation to the variables representing organizational WLB, because they are the ones transmitted by the organizations themselves within their online communication strategy. The third limitation is due to the diversity of sectors considered, having in mind that we selected companies committed to be employers of choice. However, our article makes a unique value-added contribution, throughout transparent and replicable methods within the domain of qualitative research, by expanding the discussion of WLB to attract and retain Millennials and Generation Z at work.

For the near future it would be complementary to start a study with a time dimension, which could determine evolution in WLB components and generational diversity in organizations, and to determine their relationships. Research projects that study the same WLB variables could also be carried out but in specific sectors.

## Figures and Tables

**Table 1:** Coding areas of website.

Area	Description	Example
Remote working (RW)	Possibility of working from a distance for some or all of the day.	The Goldman Sachs Group, Inc.—Based on manager approval, the following arrangements may be available to help employees meet their personal and family responsibilities: part-time schedules, job sharing, telecommuting, and alternate hours.
Sabbaticals (S)	Possibility of taking sabbatical periods from work.	David Weekley Homes—Team members have the opportunity to take a four- to six-week sabbatical after 10+ years of continuous service. This is a planned break from work that allows team members to enjoy traveling, volunteering, learning a new skill, or fulfilling a life-long dream. In addition, team members receive a grant from the company to use while on the sabbatical for activities, travel, education, etc.
Flexible hours (FH)	Possibility of flexibility of work hours within the organization, to a greater or lesser extent.	Nationwide—Flexible working: this can include options such as flexi-time, part-time, job sharing, and term-time working.
Leave (L)	Offer to increase the legally established leave for workers in the company.	NVIDIA—Offers multiple leave programs when you need to take time off from work for reasons other than vacation.
Home help (HH)	Assistance in the form of a smaller or larger amount to cover activities in the home for the candidate.	JM Family Enterprises—Dry cleaning: eco- friendly dry cleaning and shoe repair drop off and delivery service.
Assistance elderly (AE)	Economic assistance designed to cover expenses arising from care of the elderly.	KPMG LLP—Family resources: backup elder care provides access to in-home backup elder care through Bright Horizons.
Childcare (CC)	Assistance in the form of a smaller or larger amount, or provision by the company of a daycare service for employees’ children.	Kimley-Horn—Benefits at Kimley-Horn include: (…) backup childcare.
Work-life balance training (WLBT)	Training actions designed to improve the balance with personal and family life.	Recreational Equipment, Inc (REI).—Work/life employee assistance. To help find balance between work and home employees are encouraged to use the work/life program. This employee assistance program offers access to five free un-person visits per occurrence and support to help us all in your quest to live and work well.

**Table 2:** Employees’ generational diversity in BPW.

	Min (N = 59)	Max (N = 59)	Mean of Employees (%) (N = 59)	St. Deviation (N = 59)
Gen Z	0	54	2.78	10.37
Gen M	3	81	38.65	15.75
Gen X	3	55	33.56	13.17
Gen B	0	36	18.04	9.37
Gen S	0	35	0.89	4.76

**Table 3:** WLB Top Index.

Organization	Sector	Original BPW Rank	WLB Zindex	Position
JM Family Enterprises	Manufacturing and Production	51	2.38500	1°
The Goldman Sachs Group, Inc.	Financial Services and Insurance	89	1.79262	2°
Ryan LLC	Professional Services	71	1.70149	3°
Atlantic Health System	Health Care	74	1.70149	4°
Alston and Bird LLP	Professional Services	79	1.70149	5°
Alliance Data	Advertising and Marketing	82	1.70149	6°
KPMG LLP	Professional Services	29	1.68478	7°
Hyland	Information Technology	75	1.44631	8°
Genentech	Biotechnology and Pharmaceuticals	8	1.41441	9°
Nationwide	Financial Services and Insurance	53	1.02102	10°
Progressive Insurance	Financial Services and Insurance	78	1.02102	11°
Baird	Financial Services and Insurance	12	1.00431	12°
Devon Energy	Manufacturing and Production	92	0.91317	13°
Wegmans Food Markets, Inc.	Retail	2	0.88279	14°
American Fidelity Assurance Co	Financial Services and Insurance	59	0.80989	15°
Recreational Equipment, Inc.	Retail	43	0.79166	16°
David Weekley Homes	Construction	36	0.71420	17°
Workday	Information Technology	7	0.68837	18°
Baptist Health South Florida	Health Care	25	0.23270	19°
Build-A-Bear Workshop	Retail	55	0.23270	20°
